# Supplementary material for: Applying a transformer architecture to intraoperative temporal dynamics improves the prediction of postoperative delirium
Source: Commun Med (Lond). 2024 Nov 27;4:251. doi: 10.1038/s43856-024-00681-x (PMC11603037; doi:10.1038/s43856-024-00681-x)
Supplement: Supplementary file 2 — Supplementary Information [file 43856_2024_681_MOESM2_ESM.pdf]

Supplementary Information: "Applying a transformer architecture to intraoperative temporal dynamics improves the prediction of postoperative delirium"

Niklas Giesa*, Maria Sekutowicz , Kerstin Rubarth , Claudia Doris Spies , Felix Balzer , Stefan Haufe+, and Sebastian Daniel Boie+

*niklas.giesa@charite.de
+these authors contributed equally

Supplementary Figure 1. Distribution of sampling intervals

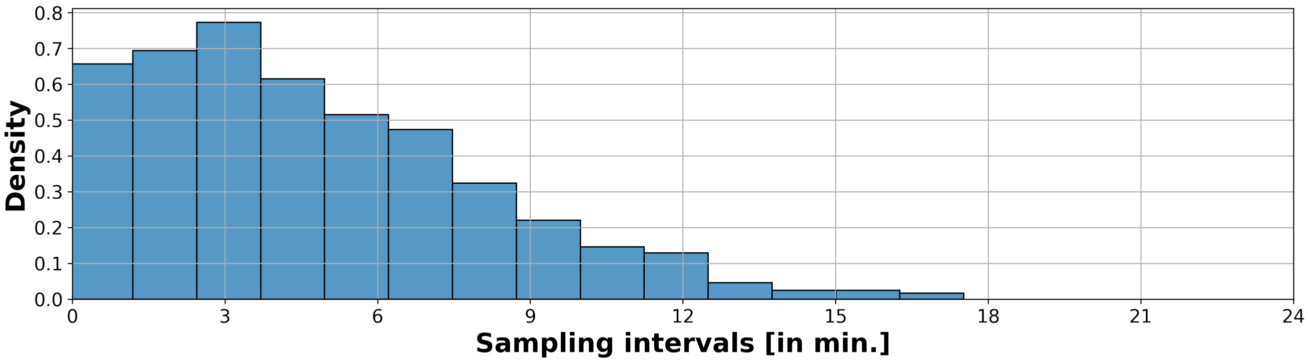

Distribution (#bins = 20) of sampling intervals measured in time differences (in minutes) between two consecutive observations. Observations are pooled from time series with respect to high frequency vital signs (blood pressure, respiratory rate, oxygen saturation, heart rate).

Supplementary Table 1. Observation windows

Observation Window	Description
[T_begin, 30]	first 30 min
[-60, T_end]	last 60 min
[30, 60]	between first 30 and 60 min
[-90, -60]	between last 90 and 60 min

Examples of observation windows defined as sub-intervals of the full intraoperative time phase $[T_{\_begin}, T_{\_end}]$ with time deltas [in minutes] added to T_begin as anesthesia induction (no prefix) or subtracted from T_end as anesthesia termination (negative prefix).

Supplementary Methods 1

We have investigated potential sampling intervals for transforming unevenly sampled time series to an equispaced time grid. Supplementary Figure 1 highlights a peak in the distribution of time distances between observations at 3 minutes for high frequency vital signs. In addition, we also aimed to measure the effect of a wider time grid. Hence, we sampled extracted longitudinal health records with both 3 minutes and 5 minutes by mean-aggregation. The selection of a sampling interval, loss function, batch size, and learning rate was included in a Grid Search[1]. Configurations for model specific hyperparameters were searched via a Hyperband search[2]. The search space outlined in Supplementary Table 4 described hyperparameters with their value ranges. Configurations for combined models were searched in an extended search space covering MLP as well as deep learning (transformer, LSTM) parameters. For LSTM models, dropout could only be applied between two stacked modules.

We integrated our hyperparameter search strategy in a 3-fold nested cross validation (CV) process[3] including different model variants (see Methods in main manuscript). Final results are provided by Supplementary Data 6. CV results for the first intraoperative 30 minutes are presented in Supplementary Figure 2. Transformer models ingesting sequential time series (TRAN_SEQ) yielded the highest validation metrics for both sampling intervals. When comparing the performance scores across sampling intervals, the differences seemed to differ marginally (see Supplementary Figure 2). However, the 3 minutes interval was evaluated with overall higher scores when summing mean scores across model variants per sampling interval. Predictive temporal signals in the time series may have been preserved by using a smaller sampling interval leading to higher validation scores.

Supplementary Figure 2. Cross-validation results

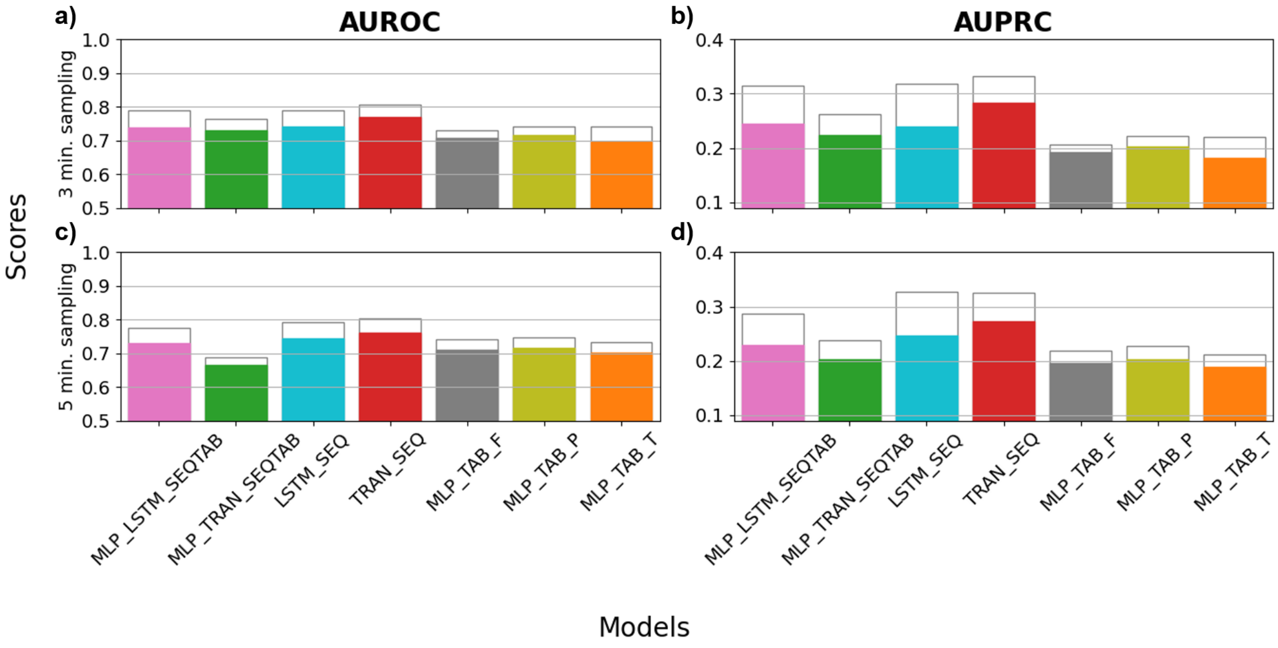

Mean performance scores (AUROC, AUPRC) per model variant calculated on outer cross-validation folds via 3x3-fold nested cross validation (CV). Validation scores are colorized per model variant, training metrics are shown as white boxes on top. All models ingest time series retrieved from the first intraoperative 30 min. (observation window [T_begin, 30]). Panels **a** and **b** show results for a 3 min. sampling interval, **c** and **d** show results for a 5 min. sampling interval.

Supplementary Figure 3. Schema of LSTM module

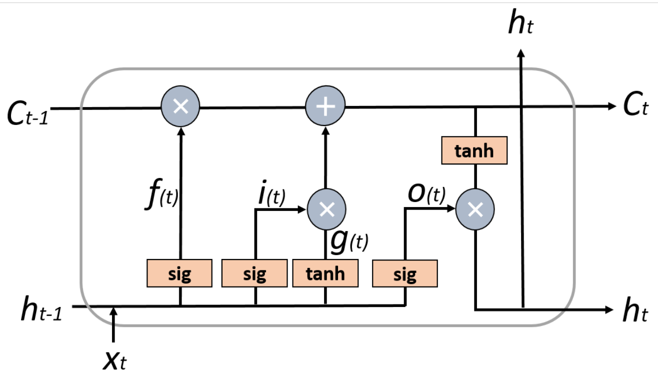

Schema of one LSTM module by Hochreiter and Schmidhuber comprising forget-, input-, and output gate defined by functions $f(t)$, $i(t)$ and $o(t)$. Module ingests last cell state C_{t-1}, last hidden state h_{t-1}, and current input x_t for the calculation of current cell state C_t and current hidden state h_t. Activation functions sigmoid logistic (sig) or hyperbolic tangent (tanh) are drawn in boxes. Pairwise product and pairwise addition are indicated by x or + in circles respectively.

Supplementary Figure 4. Schema of attention module

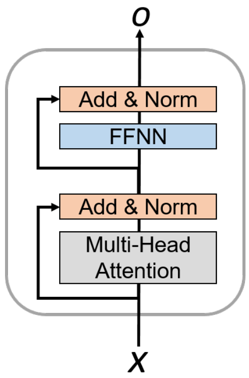

Schema of one module by Vaswani et al. used in the transformer architecture. The input x is fed into a multi-head attention mechanism. The result is added to the actual input x via a residual connection preventing vanishing gradients and normalized (Add & Norm). Afterwards, a feed-forward neural network (FFNN) is applied, results are added with the FFFN's input and normalized yielding the final output o. In a transformer model, x of one module can be comprised of input- or output embedding vectors or o of some previous module.

Supplementary Methods 2

Over the past decade, long short-term memory (LSTM) models, a form of a recurrent neural network, have gained popularity in the context of deep learning with sequential data[4]. Introduced by Hochreiter and Schmidhuber in 1997[5], the LSTM design aims to overcome the vanishing gradient problem. The problem depicts the effect of small gradients occurring at training impeding the back-propagation learning process[6]. LSTMs are designed to learn long- as well as short term temporal dependencies hampering vanishing gradients over sequential time steps[4,5,7]. Supplementary Figure 3 displays the schema of one LSTM module[5] which consumes the previous cell state C_{t-1} and the previous hidden state h_{t-1} from the prior module. On the basis of the current input x_t and h_{t-1}, the forget gate $f(t)$ decides if parts of C_{t-1} are updated for calculating the new cell state C_t. Function $\tilde{f}(t)$ from the equation 1 is used in $f(t)$ with a logistic sigmoid function[8] (sig), the output is pairwise multiplied with C_{t-1}.

$$\tilde{f}(t) = \sigma(W_f \times [h_{t-1}, x_t] + b_f) \tag{1}$$

- σ: either a logistic sigmoid (sig)- or a hyperbolic tangent (tanh) function
- $W_f \in \mathbb{R}^{k\times d}$: weighting matrix that is learned via training
- $b_f \in \mathbb{R}^k$: bias vector that is learned via training
- k: superscript as number of hidden states $|h|$
- d: superscript as number of input features $|x|$

For the realization of the input gate $i(t)$, $\tilde{f}(t)$ is configured with a sig activation. The function $g(t)$ uses a hyperbolic tangent[8] (tanh)- instead of a sig activation function in $\tilde{f}(t)$ and calculates candidates values for the current cell state C_t. Outputs of $i(t)$ and $g(t)$ are then pairwise multiplied and added to the output of the previous applied forget gate $f(t)$. On the one hand, this result contributes directly to the current cell state C_t. On the other hand, the result is fed into a tanh activation function and pairwise multiplied with the output gate $o(t)$ that uses $\tilde{f}(t)$ with sig activation. The resulting pairwise product defines the current hidden state h_t (see Supplementary Figure 3). A simplified version of LSTM networks are called gated recurrent units (GRUs) that focus on less gating mechanisms, thus demanding fewer trainable parameters saving training expenses[9].

In 2017, Vaswani et al.[10] described the transformer (TRAN) model as a network architecture based on the attention mechanism that was introduced by Bahdanau et al. in 2014[11]. Thier aim was to decrease computational time caused by LSTM models when applied to large complex datasets. In a seq2seq setting, an encoder translates a sequential input into a latent space. A decoder converts the latent representation back to a sequential output[12]. Transformers aim to improve computational efficiency via parallelization using self-attention in encoder and decoder modules[5,10,12,13]. Vaswani et al. describe their attention computation as "Scaled Dot-Product Attention" based on the concept of queries Q, keys K, and values V[10].

$$Attention(Q,K,V) = softmax(\frac{QK^T}{\sqrt{d_k}})V \tag{2}$$

- Q: set of output values predicted as embedding vectors
- K, V: set of given input values as embedding vectors
- d_k: dimension of Q and K

In equation 2, the numerator inside the $softmax$ function calculates the dot product of Q with K. The denominator divides the dot product by $\sqrt{d_k}$ in order to limit the growth of the term inside the $softmax$ function. This mechanism prevents vanishing gradients as a result of outputs with little decent caused by the $softmax$ activation applied for large inputs[10].

$$MultiHead(Q,K,V) = Concat(head_1,...,head_n)W^O \tag{3}$$

$$head_i = Attention(QW_i^Q, KW_i^K, VW_i^V) \tag{4}$$

In equation 4, embedding vectors Q, K, and V are parameterized with trainable weighting matrices W_i^Q, W_i^K, and W_i^V respectively and fed into the attention mechanism. One single parameterized attention is called attention head ($head_i$). In the transformer model, multiple attention heads are concatenated and weighted with the trainable matrix W_O (see Equation 3).

In the seq2seq setting, Vaswani et al. implemented $1...n$ modules (Supplementary Figure 4) for training the encoder and decoder using the same sequence as in- and output for the multi-head attention mechanism. This approach is also called self-attention. For the difference between the encoded and the decoded sequence, the authors propose the so-called cross-attention. In cross-attention, the latent representations of the in- and output sequence are fed into the multi-head attention mechanism[10].

During the processing of all sequential data at a time, positional information is lost in the attention mechanism. Hence, a positional encoding is usually applied to the embedding vectors for retaining this information[10,14]. Work that based on Vaswani et al. designed popular variations of transformer architectures like BERT or GPT-3[15,16]. For our study, we have used the implementations of LSTM and TRAN modules provided by Pytorch[17].

Supplementary Table 2. Constructed model variants

Model	Data	Abbreviation
Long short-term memory + multi-layer perceptron	Sequential and tabular	MLP_LSTM_SEQTAB
Transformer + multi-layer perceptron	Sequential and tabular	MLP_TRAN_SEQTAB
Long sort-term memory	Sequential	LSTM_SEQ
Transformer	Sequential	TRAN_SEQ
Multi-layer perceptron	Tabular	MLP_TAB_F
Multi-layer perceptron	Tabular	MLP_TAB_P
Multi-layer perceptron	Tabular	MLP_TAB_T

Trained model variants along with data input types and abbreviation used throughout the manuscript where MLP: Multi-layer perceptron, LSTM: long short-term memory, TRAN: transformer, SEQ: sequential, TAB: tabular, and post-fixes _F: time-features like Wavelets, _P: summary statistics like percentiles, _T: transposed data (one column per time step).

Supplementary Table 3. Final model parameters

Model	MLP	LSTM	Transformer
MLP_LSTM_SEQTAB	12 neurons, 3 layer	10 hidden neurons, 3 layers	-
MLP_TRAN_SEQTAB	10 neurons, 2 layers	-	2 encoder layers, 6 neurons, 4 layers
LSTM_SEQ	-	12 hidden neurons, 3 layers	-
TRAN_SEQ	-	-	1 encoder layers, 6 neurons, 2 layers
MLP_TAB_F	8 neurons, 4 layers	-	-
MLP_TAB_P	6 neurons, 4 layers	-	-
MLP_TAB_T	6 neurons, 4 layers	-	-

Parameters as number of neurons and layers for each model variant.

Supplementary Table 4. Hyperparameter search space

Model	Hyperparameter	Value Options	Algorithm
All	loss function	{"focal loss", "weighted binary cross entropy"}	Grid Search
	sampling interval	{"5 min.", "3 min."}	
	learning rate	{1e-5, 1e-4}	
	batch size	{32, 64}	
MLP	#hidden layers	{4, 6, 8}	Hyperband Search
	#neurons per layer	{2, 4, 8, 16}	
LSTM	#stacked modules	{1, 2, 3}	
	#hidden neurons	{4, 6, 8, 10, 12}	
	dropout	{0, 0.1, 0.2}	
TRAN	#hidden dimensions	{16, 32, 64, 128}	
	#attention heads	{1, 2}	
	#hidden layers	{4, 6, 8}	
	#neurons per layer	{2, 4, 8, 16}	

Search space is determined by the value options and hyperparameters per model variant. We configured a multi-layer perceptron (MLP), long short-term memory (LSTM), and transformer (TRAN).

Supplementary Figure 5. Training performance

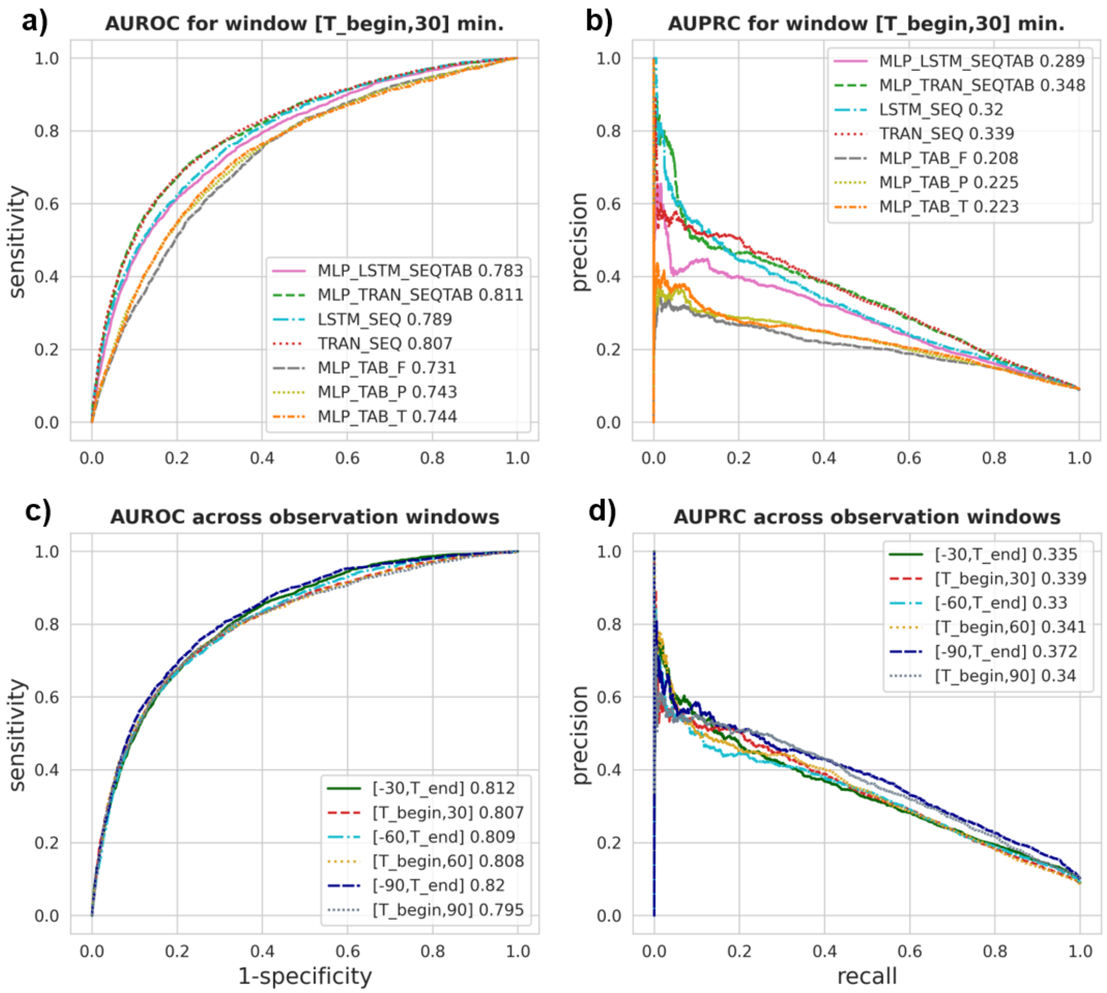

Training AUROC and AUPRC curves across model variants, panels **a** and **b**, or observation windows, panels **c** and **d**. Models were implemented as MLP (MLP), transformer (TRAN), LSTM (LSTM), or a combination of these. Models ingest sequential (SEQ) and / or aggregated tabular (TAB) data. For **b** and **c**, all models were implemented as TRAN ingesting SEQ. Observation windows lay within the intraoperative phase from T_begin to T_end. Either the first (no prefix) or the last (dash as prefix) 30, 60, or 90 min. were used. Random classification levels were at 0.5 and 0.09 for AUROC and AUPRC, respectively.

Supplementary Methods 3

Training performance in form of AUROC and AUPRC curves are outlined in Supplementary Figure 5. Trends that were observed during CV and the final testing (see Results in main manuscript) were also present in the training curves. AUPRC scores overlapped between deep learning model variants (those comprising LSTM or TRAN). The difference in performances between stand-alone MLP models ingesting aggregated tabular (TAB) data and deep learning models was pronounced. Similar to results drawn from the previous observation window analysis, training curves did not differ visually between observation windows. We used regularization techniques like dropout or early stopping via nested CV[2,18]. Results did not highlight a pronounced problem of overfitting.

Supplementary Figure 6. Cohort inclusion criteria

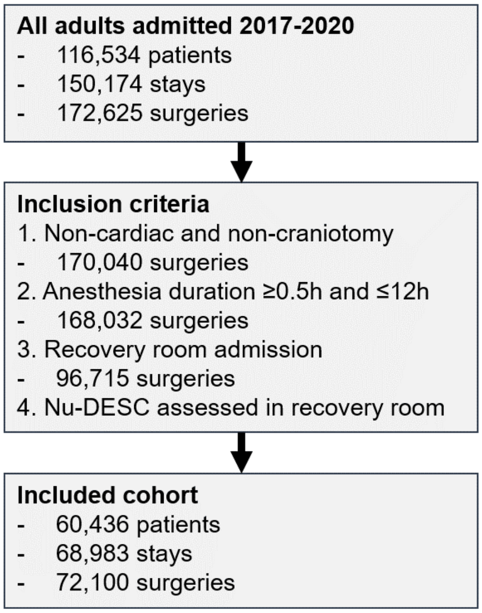

Inclusion criteria for the cohort selection. The included cohort consists of adult patients admitted between 2017-2020 with an anesthesia duration of at least half an hour and a maximum of 12 hours. Surgeries that could be assigned to at least one Nu-DESC assessed in the recovery room were included.

Supplementary Results 1

Supplementary Figure 6 displays applied inclusion criteria for the definition of our study cohort. We excluded cardiac surgeries and craniotomy procedures due to the overall high prevalences of postoperative delirium (POD) for such patients[19–21]. The cohort was also limited to cases with expected general anesthesia (documented a priori) between 0.5 and 12 hours and to recovery room admissions. Hereby, we focused on a population being neither too strong or too little susceptible to suffering from POD[22]. Over the past years, our clinical institution has established the usage of the observational nursing delirium screening scale (Nu-DESC) for POD assessment in the recovery room. We used the Nu-DESC for the definition of our binary POD target variable Y (see Methods in main manuscript). Characteristics of our included patients are presented in Supplementary Table 5. Further descriptive statistics are included in Supplementary Data 5.

Supplementary Table 5. Patient characteristics

Domain	Variable	All surgeries	POD positives	POD negatives
Counts	#patients	60,436	5,673	54,763
	#cases	68,983	5,843	63,140
	#surgeries	72,100	5,963	66,137
	#surgeries per case	1.36 ± 0.79, [1, 1, 1]	1.33 ± 0.75, [1, 1, 1]	1.37 ± 0.79, [1, 1, 1]
	#prev. surgeries	0.50 ± 1.26, [0, 0, 1]	0.54 ± 1.27, [0, 0, 1]	0.50 ± 1.26, [0, 0, 1]
	#Nu-DESC scores	1.20 ± 0.54, [1, 1, 1]	1.27 ± 0.69, [1, 1, 1]	1.19 ± 0.53, [1, 1, 1]
Durations	anesthesia [hours]	2.07 ± 1.26, [1, 1, 2]	2.51 ± 1.36, [1, 2, 3]	2.03 ± 1.24, [1, 1, 2]
	recovery room [hours]	3.13 ± 3.09, [1, 1, 2]	4.65 ± 7.54, [1, 2, 3]	2.99 ± 2.63, [1, 1, 2]
	hospitalization [days]	8.58 ± 5.99, [2, 4, 8]	11.45 ± 7.63, [3, 6, 11]	8.31 ± 5.78, [2, 4, 7]
	transfer to recovery room [min]	7.34 ± 4.92, [4, 6, 9]	7.57 ± 5.45, [4, 6, 9]	7.35 ± 5.21, [4, 6, 9]
	extubation to Nu-DESC [min]	37.39 ± 26.83, [13, 31, 57]	37.67 ± 40.46, [10, 20, 50]	52.56 ± 43.12, [16, 41, 76]
Demographics	age [years]	53.53 ± 18.41, [37, 55, 69]	60.28 ± 18.88, [46, 63, 76]	52.93 ± 18.22, [37, 54, 68]
	bmi [kg/m^2]	26.61 ± 5.57, [22, 25, 29]	26.59 ± 5.49, [22, 25, 29]	26.61 ± 5.58, [22, 25, 29]
	gender (f/m)	53.19% female	51.33% female	53.60% female
Scores	N class[1]	4.24 ± 1.13, [3, 5, 5]	4.28 ± 1.10, [4, 5, 5]	4.24 ± 1.14, [3, 5, 5]
	ASA status	2.14 ± 0.72, [2, 2, 3]	2.35 ± 0.72, [2, 2, 3]	2.12 ± 0.72, [2, 2, 3]
	RASS	0.58 ± 1.01, [0, 0, 1]	0.98 ± 1.33, [0, 1, 2]	0.51 ± 0.92, [0, 0, 1]
	frailty index	1.05 ± 1.03, [0, 1, 2]	1.25 ± 1.07, [0, 1, 2]	1.03 ± 1.02, [0, 1, 2]
Vital Signs	Spo2	97.94 ± 2.26, [97, 98, 100]	97.45 ± 2.51, [96, 98, 99]	$97.99.12 \pm 2.23$, [97, 89, 100]
	heart rate	75.62 ± 14.20, [65, 74, 84]	75.29 ± 14.17, [65, 74, 83]	75.65 ± 14.20, [66, 74, 84]
	IBP[2] systolic	118.72 ± 19.62, [108, 116, 126]	122.21 ± 17.93, [110, 119, 131]	118.13 ± 19.83, [108, 116, 125]
	IBP diastolic	60.04 ± 12.84, [54, 59, 65]	58.59 ± 11.19, [52, 58, 65]	60.28 ± 13.09, [54, 59, 65]
	NIBP[3] systolic	133.65 ± 21.07, [118, 131, 147]	135.44 ± 22.05, [119, 134, 150]	133.48 ± 20.97, [118, 131, 144]
	NIBP diastolic	77.18 ± 12.14, [59, 77, 85]	76.52 ± 12.16, [68, 76, 84]	77.24 ± 12.39, [69, 77, 85]
Anesthesia	balanced	50.33%	47.01%	50.63%
	TIVA[4]	43.25%	54.18%	42.26%
	spinal	5.21%	0.79%	5.60%
Comorbidities	dementia	2.88 ± 3.79, [0, 1, 4]	2.97 ± 3.94, [0, 2, 4]	2.87 ± 3.76, [0, 1, 4]
	parkinson	2.47 ± 3.30, [0, 1, 4]	2.72 ± 3.36, [0, 1, 5]	2.38 ± 3.28, [0, 1, 3]
	stroke	2.38 ± 2.83, [0, 1, 4]	3.00 ± 3.24, [0, 1, 5]	2.354 ± 2.27, [0, 1, 4]
	CHD[6]	2.27 ± 4.18, [0, 1, 3]	1.98 ± 3.07, [0, 1, 2]	2.32 ± 4.32, [0, 1, 3]
Past Surgeries	abdominal	6.47 ± 4.29, [0, 1, 3]	2.76 ± 5.71, [0, 1, 3]	6.80 ± 5.30, [0, 1, 3]
	otorhinolary	2.28 ± 1.67, [0, 1, 3]	1.56 ± 1.53, [0, 1, 2]	2.31 ± 1.68, [0, 1, 3]
	endocrine	1.11 ± 1.31, [0, 1, 1]	1.06 ± 1.21, [0, 1, 2]	1.12 ± 1.33, [0, 1, 1]
	maxillofacial	1.04 ± 1.67, [0, 1, 1]	0.98 ± 1.53, [0, 0, 1]	1.04 ± 1.68, [0, 1, 1]

Characteristics based on preoperative information. Summary statistics are shown for all surgeries, for surgeries defined as POD positive (one Nu-DESC > 0), and for surgeries defined as POD negative (all Nu-DESC = 0). Counts # are displayed as absolute numbers. Statistics for numerical variables are displayed as mean±std, [1st, 2nd, 3rd] quartile. Comorbidities and past surgeries are included with statistics for the numbers of previous encoded occurrences.

[1]surgery urgency (urgent 0 - elective 5), [2]invasive blood pressure, [3]non-invasive blood pressure, [4]total intravenous anesthesia, [5]peripheral arterial disease, [6]coronary heart disease

Supplementary Table 6. Time-static correlations

Feature	Description	p-value	Coefficient
curr_dem	Diagnose of dementia	2.29e-197	+0.136
age	Patient age	1.16e-138	+0.114
praem_oplen	Estimated surgery length	1.68e-37	+0.110
asa	ASA physical status classification	1.56e-64	+0.102
met_equi	Metabolic equivalents of task	2.62e-15	-0.093
anes_totaliv	Total invasive anesthesia	6.80e-60	+0.074
hst_visual_op	Previous ophthalmology surgery	1.17e-04	-0.069
hst_hear_op	Previous otorhinolaryngology surgery	1.17e-04	-0.069
anes_spinal	Spinal anesthesia	2.10e-43	-0.063
hst_skin_op	Previous skin surgery	4.19e-06	-0.058
prev_adm	#of previous admissions	1.85e-18	+0.040
prev_surg	#of previous surgeries	2.22e-11	+0.030
op_n	Surgery urgerncy index	3.97e-07	+0.030
body_length	Body length	2.05e-05	-0.029
anes_epidu	Epidural anesthesia	1.21e-08	-0.026
gender	Gender (1=female)	8.57e-08	-0.024
anes_balanced	Balanced ansesthesia	2.06e-06	-0.022
anes_plex	Plexus anesthesia	4.53e-06	-0.021
curr_alcdis	Diagnosed alcoholic disorder	8.92e-06	+0.020
curr_smok	Frequently smoking	9.30e-05	+0.018

Spearman's rank correlation coefficients for time-static features (Feature) sorted descending by the absolute value of coefficients. Top 20 significant features are included. Positive or negative effect directions are indicated (+/-). All features were statistically significant according to a FDR corrected alpha level.

Supplementary Figure 7. P-values for mixed linear effects

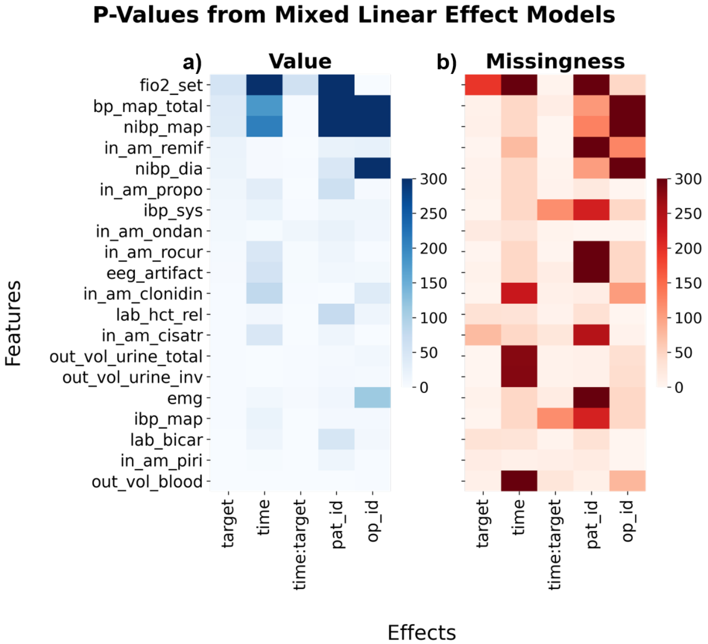

Log-transformed (natural logarithm) P-Values of mixed linear effect models (MLEMs) linking means of time-dynamic features within analysis windows to POD presence (target), analysis interval (time), and time x target interactions (time:target), modeled as fixed effects. A correction for the relationship of patients having multiple surgeries was introduced by nested random effects (not shown). Time encodes three consecutive 30-minutes non-overlaping interoperative analysis windows relative to the beginning of the surgery (T_begin). Model coefficients for fixed effects (columns) are shown per feature (rows). Models were fitted on normalized feature values (left/blue), shown in panel **a**, or corresponding missingness rates (right/orange), displayed in panel **b**. Effect directions are indicated with +/- signs drawn in red for effects that are statistically significant according at an FDR corrected alpha level of 0.05. Results are shown for 20 features exhibiting largest log-transformed P-Value for POD onset (target). N = 48,348 patients in the training set.

Supplementary Results 2

We aimed to investigate the effect of observation windows with varying lengths and positions on model performances. During our CV process, the transformer model (TRAN_SEQ) yielded first promising validation metrics. Hence, we trained TRAN_SEQ models for six different observation windows with bootstrapped data. Windows were holding either the first or the last 30, 60, and 90 minutes of multivariable intraoperative time series (see Methods in main manuscript). By using a 3 minutes sampling interval, we created sequences with lengths of 10, 20, and 30 respectively. We did not use padding or other techniques to extend sequences due to potentially biased estimates[23]. Thus, we included 72,100, 69,908, and 65,814 surgeries for the analysis with minimum lengths of 30, 60, and 90 minutes.

Supplementary Table 7 and Supplementary Figure 8 show testing performance metrics for TRAN_SEQ models applied on data from different observation windows. When considering AUROC as a metric, the model ingesting the last intraoperative 60 minutes ([-60, T_end]) achieved the best result with a mean of 0.778 (95%-CI [0.776, 0.779]). The fitted model yielded a mean sensitivity and a mean specificity of 0.672 and 0.753 respectively at the threshold were their sums maximize. We could observe an overlap of confidence intervals (CIs) regarding AUROC between this ([-60, T_end]) observation window and others ([T_begin,30], [T_end,60], [-30,T_end]). The TRAN_SEQ model that used the first 30 minutes ([T_begin, 30]) achieved the highest AUPRC with a mean of 0.330 (95%-CI [0.328, 0.340]). This model reached a mean sensitivity of 0.711 at 0.711 specificity. CIs of AUPRC scores that were performed by other observation windows did not overlap with [T_begin, 30]. Supplementary Data 2 in Supplementary B includes additional performance metrics retrieved on the bootstrapped testing set.

Supplementary Figure 9 illustrates the distributions of AUROC and AUPRC scores across observation windows in addition to statistical testing results. Overall, there were just a few significant results when comparing AUPRC metrics across observation windows. There were no significant differences in AUROCs between observation windows (bottom left in Supplementary Figure 9). As our previous findings suggested, we found statistical evidence that AUPRCs retrieved from the first intraoprative 30 minutes ([T_begin, 30]) differed from three distinct observation windows ([T_begin, 60], [T_begin, 90], [-90, T_end]) (bottom right in Supplementary Figure 9).

Our analysis tested various observation windows training our best model variant. Results did not clearly highlight any intraoperative time window with superior predictive power. The consumption of the first 30 minutes of a surgery was evaluated with overall higher metrics that were susceptible to class imbalance.

Supplementary Figure 8. Prediction threshold curves for window analysis

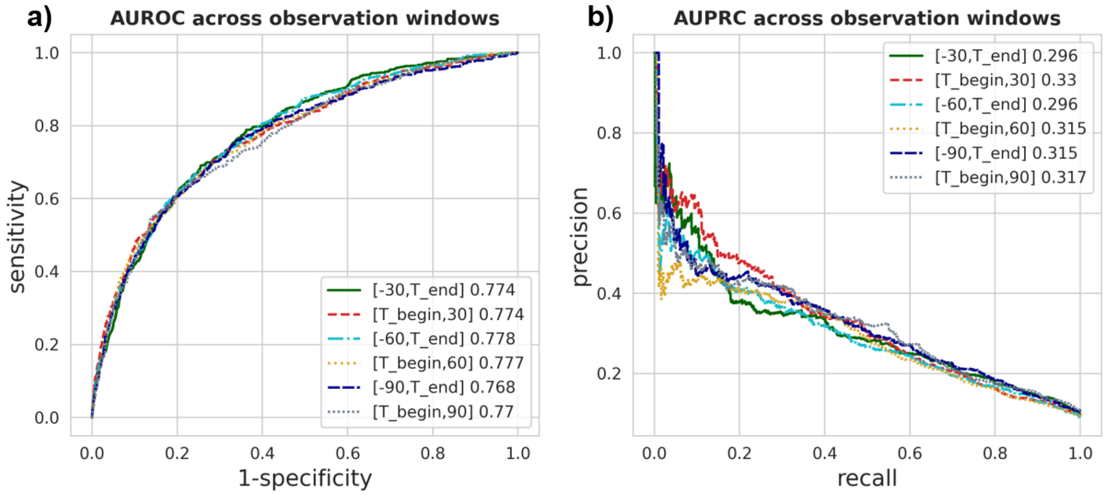

Testing AUROC and AUPRC curves shown in panel **a** and **b** for different observation windows fed into a sequential transformer model (TRAN_SEQ). Observation windows lay within the intraoperative time phase from T_begin to T_end covering either the first (no prefix) or the last (dash as prefix) 30, 60, or 90 min. Random classification levels were at 0.5 and 0.09 for AUROC and AUPRC, respectively.

Supplementary Table 7. Performance metrics per observation window

Window	AUROC	AUPRC
[T_begin,30]	0.774 [0.772, 0.787]	0.330 [0.328, 0.340]
[T_begin,60]	0.777 [0.776, 0.779]	0.315 [0.312, 0.318]
[T_begin,90]	0.770 [0.768, 0.771]	0.317 [0.314, 0.320]
[-30,T_end]	0.774 [0.773, 0.776]	0.296 [0.293, 0.298]
[-60,T_end]	0.778 [0.776, 0.779]	0.296 [0.293, 0.299]
[-90,T_end]	0.768 [0.766, 0.770]	0.315 [0.312, 0.318]

Metrics were calculated on 1000x bootstrapped testing set. Results are reported as mean [95%-CI]. All models were implemented as transformers ingesting sequential data (TRANS_SEQ). Observation windows lay within the intraoperative time phase from T_begin to T_end. Either the first (no prefix) or the last (dash as prefix) 30, 60, or 90 minutes of the intraoperative time phase were used with a 3 minute sampling interval

Supplementary Figure 9. Performance confidence for window analysis

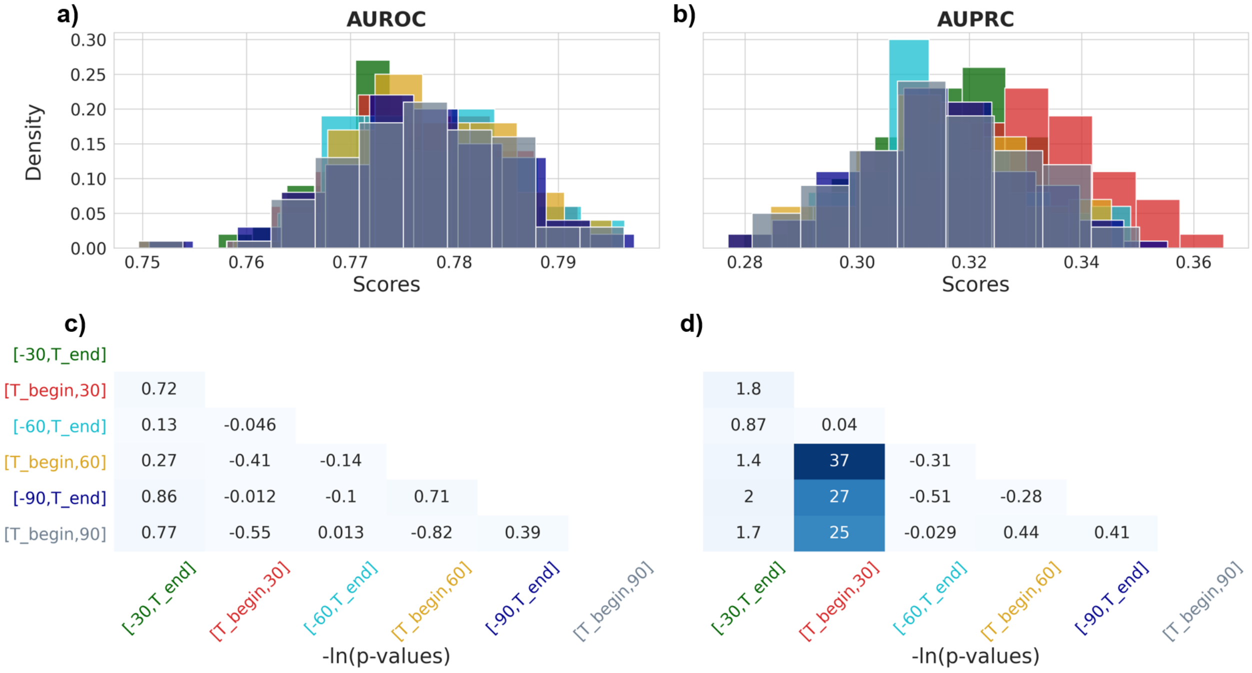

Final validation scores across observation time windows calculated on a 1000x bootstrapped testing set. Distribution of AUROC and AUPRC scores are shown in panels **a** and **b**. A corrected pairwise Student's T-test was used to calculate p-values for the comparison of performance scores between two observation windows. The –ln(p-value) was retrieved for AUROC and AUPRC scores shown in panels **c** and **d**. All observation windows provided sequential data between the start (T_begin) and end (T_end) of the intraoperative phase for a transformer model (TRANS_SEQ). Either the first (no prefix) or the last (dash as prefix) 30, 60, or 90 min. of the intraoperative time phase were used.

Supplementary Figure 10. Prediction threshold curves for clinical baselines

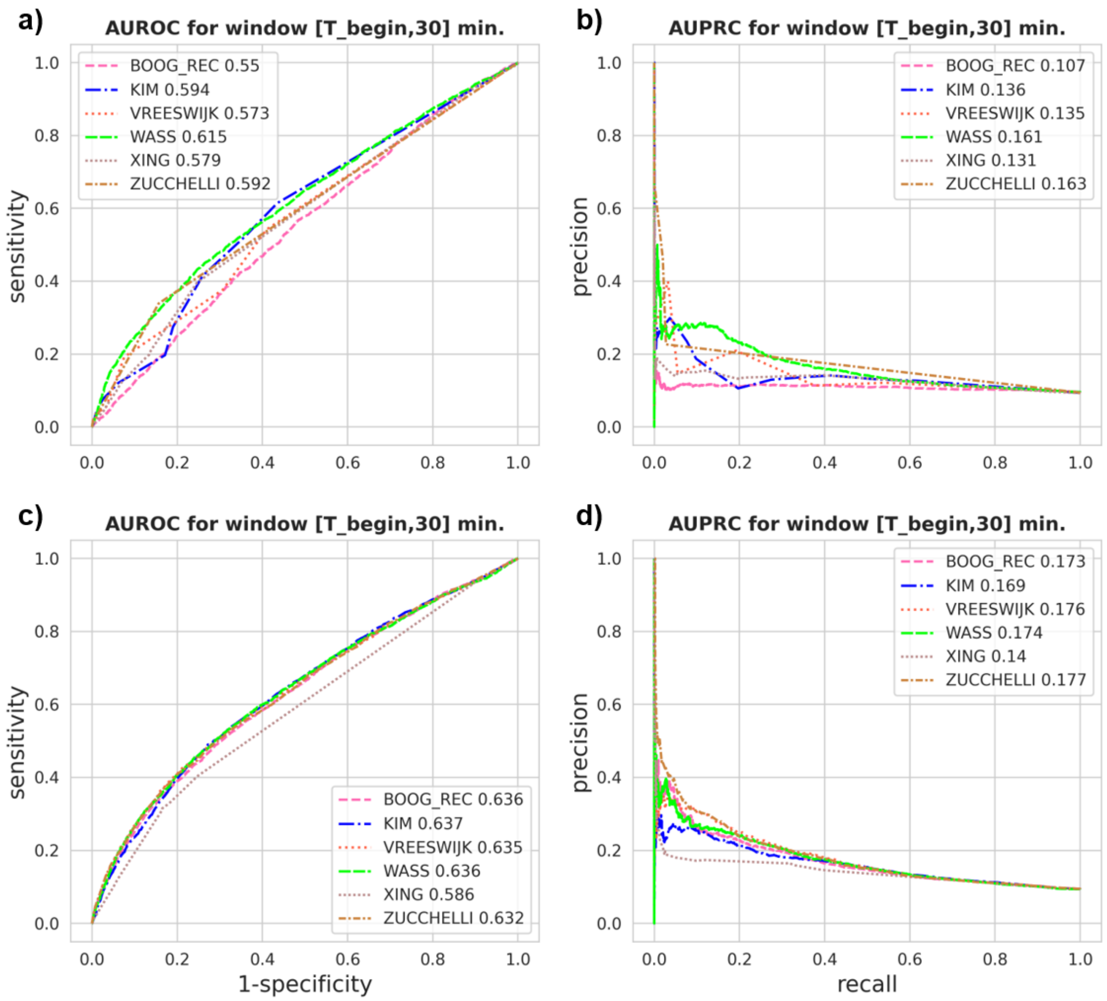

Testing AUROC and AUPRC curves for baseline models either with published, see planes **a, b**, or retrained, see planes **c, d**, coefficients. All models ingested the first intraoperative 30 min. ([T_begin, 30]). Models were published by Boogaard et al. (boog_rec), Wassenaar et al. (wass), Kim et al. (kim), Vreeswijk et al. (reeswijk), and Zuccheli et al. (zuccheli). Random classification levels were at 0.5 and 0.09 for AUROC and AUPRC respectively.

Supplementary Results 3

Various POD prognostic models have been published over the past years[19]. We have searched for openly accessible work that could be generalized for delirium classifications not necessarily constrained to a perioperative setting. Boogaard et al. and Wassenaar et al. developed delirium prediction models for intensive care patients using predictors recorded early after hospital admission[24, 25]. Vitals signs, like blood pressure, were extracted from our clinical information systems. Boogaard et al. published after their first model version re-calibrated coefficients that we used for our implementation[25]. Kim et al. constructed a POD risk score that divides patients into low risk- or high risk groups combining preoperative patient characteristics that were documented during our anesthesia consent meeting[26]. Similar to Kim et al., Vreeswijk et al. suggested a simple risk score for delirium evaluation in a general hospital setting weighting predisposing factors[27]. Zucchelli et al. validated and developed a risk score on elderly patients in the emergency department[28]. Xing et al. focused on POD occurrence after hip surgeries[29]. Additional authors have not been willing to share their models for validation purposes[30–33]. We extracted corresponding variables from our clinical systems and median-aggregated numerical feature values. Details on feature encodings for baseline models are cited in Supplementary Data 6.

All studies above have used a multi-variable logistic regression (LR) approach trained on relatively small sample sizes in

Supplementary Table 8. Performance metrics for clinical baselines

Model	Coefficients	AUROC	AUPRC
boog_rec	applied	0.550 [0.549, 0.550]	0.107 [0.106, 0.107]
wass	applied	0.615 [0.614, 0.616]	0.161 [0.160, 0.161]
kim	applied	0.594 [0.594, 0.595]	0.136 [0.136, 0.137]
vreeswijk	applied	0.573 [0.572, 0.574]	0.135 [0.135, 0.136]
zucchelli	applied	0.592 [0.591, 0.592]	0.163 [0.163, 0.164]
xing	applied	0.579 [0.578, 0.580]	0.131 [0.130, 0.131]
boog_rec	retrained	0.636 [0.635, 0.636]	0.173 [0.172, 0.174]
wass	retrained	0.636 [0.635, 0.637]	0.174 [0.173, 0.174]
kim	retrained	0.637 [0.636, 0.637]	0.169 [0.168, 0.169]
vreeswijk	retrained	0.635 [0.634, 0.636]	0.176 [0.175, 0.177]
zucchelli	retrained	0.632 [0.632, 0.633]	0.177 [0.177, 0.178]
xing	retrained	0.586 [0.585, 0.587]	0.140 [0.140, 0.141]

Performance metrics per baseline model variant (Model) calculated on 1000x bootstrapped testing set. Models were either applied with published coefficients (applied) or coefficients were fitted again (retrained). All models were applied on time series retrieved from the first intraoperative 30 min ([T_begin, 30]) and static features. Results are reported as mean [95%-CI].

the range of 200-500 patients. Authors have published data wrangling strategies, model coefficients, intercepts, or scoring thresholds. Most of them identified static-features like age, history of delirium, alcohol abuse, and cognitive or physical impairments as important predictors[19,24–28]. Time-dynamic features that were seldom used relied mostly on laboratory values or vital signs[24–26]. We have choosen these baseline models due to their simple inputs that were highly available in our extracted clinical records. All baseline models can be downloaded with usage notes via our GitHub page[34]. Results from applying and retaining the introduced five baseline models by Boogaard et al. (boog_rec), Wassenaar et al. (wass), Kim et al. (kim), Vreeswijk et al. (reeswijk), and Zuccheli et al. (zuccheli) are shown in Supplementary Figure 10 and Supplementary Table 8.

The retrained model by Kim et al. yielded the highest mean AUROC of 0.637 (95% CI [0.636, 0.637]). Mean sensitivity was at 0.490 and mean specificity was at 0.728. The model did not clearly outperform retrained models by Wassenaar et al., Boogaard et al., and Vreeswijk et al. due to the overlap in CIs for AUROCs. In Supplementary Table 8, the model with retrained coefficients by Zucchelli et al. reached the highest mean AUPRC of 0.177 (95% CI [0.177, 0.178]). Mean values were at 0.407 and 0.802 for sensitivity and specificity respectively. Predictions by Vreeswijk et al. achieved similar AUPRCs with a mean of 0.176. We calculated sensitivities and specificities for the threshold where their sum maximizes.

Baseline models failed to reach high performance metrics in our intraoperative POD setting. In contrast to model authors, we included a large heterogeneous population of over 60,000 patients. Results suggest that simple LR models predicting POD on the primarily basis of static features were not suitable for a complex intraoperative setting at our clinical institution.

Supplementary Figure 11. Prediction threshold curves for GRU-D

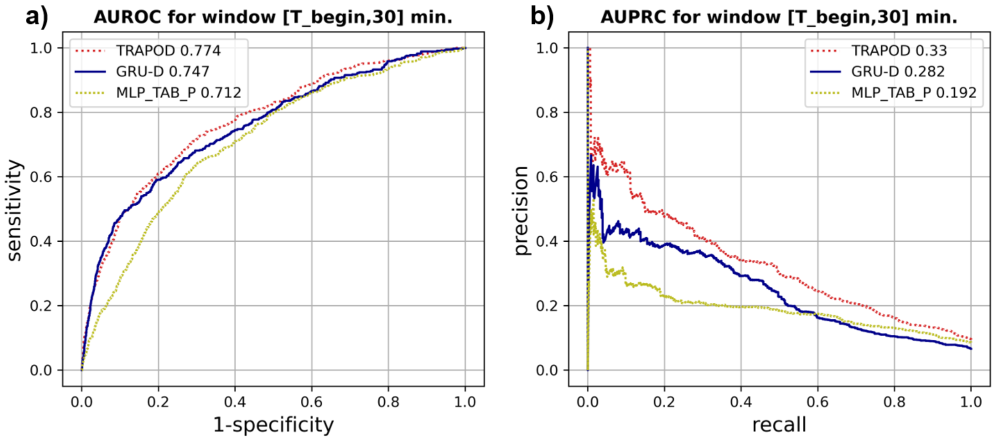

Model performance of GRU-D trained on the first intraoperative 30 minutes ([T_begin,30]) time series. Performance was evaluated with AUROC and AUPRC curves included in panels **a** and **b**, respectively. We display performance of TRAPOD and our best MLP model trained with summary statistics (MLP_TAB_P). Random classification levels were at 0.5 and 0.09 for AUROC and AUPRC, respectively.

Supplementary Results 4

In 2018, Che et al. introduced a modified version of a GRU model (namely GRU-D) that explicitly learns patterns jointly with a clinical prediction task[35] (see section LSTM and Transformer). The authors encoded unobserved information in clinical time series with two mechanisms denoted as masking and time-interval.

Let $X = (x_1, x_2, ..., x_t)^T$ be one clinical time series of our model input space potentially holding a value $x \in \mathbb{R}$ at a time-index t. The so-called masking vector $m_t \in (0,1)$ encodes for each t, if the corresponding value is missing (1) or present (0). This approach can be seen equivalent to introducing binary missing-indicator variables (MIVs)[36] that we also use in our models to enhance feature spaces (see Methods in main manuscript). The time-interval mechanism captures the time delta between two sequential non-missing time series values, so encoding the duration of a parameter's absence. Assuming s_t represents the timestamp of each t, the time-interval vector δ_t can be defined as $\delta_t = s_1 - s_2$ if $t > 1, m_{t-1} = 1$ meaning that the last value is present, $\delta_t = s_1 + \delta_{t-1}$ if $t > 1, m_{t-1} = 0$ for last value being absent, and else $\delta_t = 0$ for the initial element in X[35].

Che et al. enhanced the traditional GRU architecture by parameterized decay rates that take advantage of the missingness representations in m_t and δ_t. So-called decay rates should learn the tendency of clinical parameters to converge towards a default value when missing over a certain period of time. The decay rate γ at time index t was defined as $\gamma_t = \exp\{-max(0, W_\gamma \delta_t + b_\gamma)\}$ with W_γ and b_γ as trainable parameters. Values v_γ for γ reside between 0 and 1 where 1 indicates dependency on the past measured value solely and $v_\gamma \neq 1$ indicates convergence behaviour. In GRU-D, the input X is mathematically combined with the masking vector m_t and the decay rate γ_t so that unobserved values are described as converging towards the empirical mean of the training data from the last known observation onwards (see Che et al.[35]). The authors claim higher fidelity of this method in contrast to last observation carried forward (LOCF) where the last known value lo is always used for temporal imputation[37]. In addition to the input X, γ is also applied to the model's hidden states learning described convergence patterns for a longer period of time. The feature space concatenates matrices holding all X (I), m (M), δ (D), and lo (LO) to a 4-dimensional matrix. Hence, GRU-D learns decay rates through the missingness context and, after configuration, with respect to a target variable.

In our study, we selected 171 variables (see Table 3 in Methods of main manuscript) including four composite features, 19 additionally derived drug values, and 148 pre-selected parameters. Since GRU-D's feature space holds all missingness information, we did not add MIVs like we did in the main manuscript. Time series were retrieved for the first intraoperative 30 minutes ([T_begin, 30]) with a three minutes sampling interval. Dimensions for all matrices (I, M, D, LO) were at N (#samples) $\times$ 171 (#features) $\times$ 30 (sequence length) with N=57,680 for the training -, N=14,420 for the testing set. GRU-D comprises update-, reset-, and hidden gates that are all parameterized with 171 for the input-, hidden-, and output dimension per gate. Decay rates for either model inputs (γ_X) or hidden states (γ_h) were temporally learned per single feature. In the end, a trainable linear layer summarizes 171 inputs into one single output per sequence for the classification of POD. We configured a weighted binary cross entropy loss function[38], trained for 120 epochs with a learning rate of 1e-4 and early stopping (patience=10)[39].

Supplementary Figure 12. GRU-D decay rates

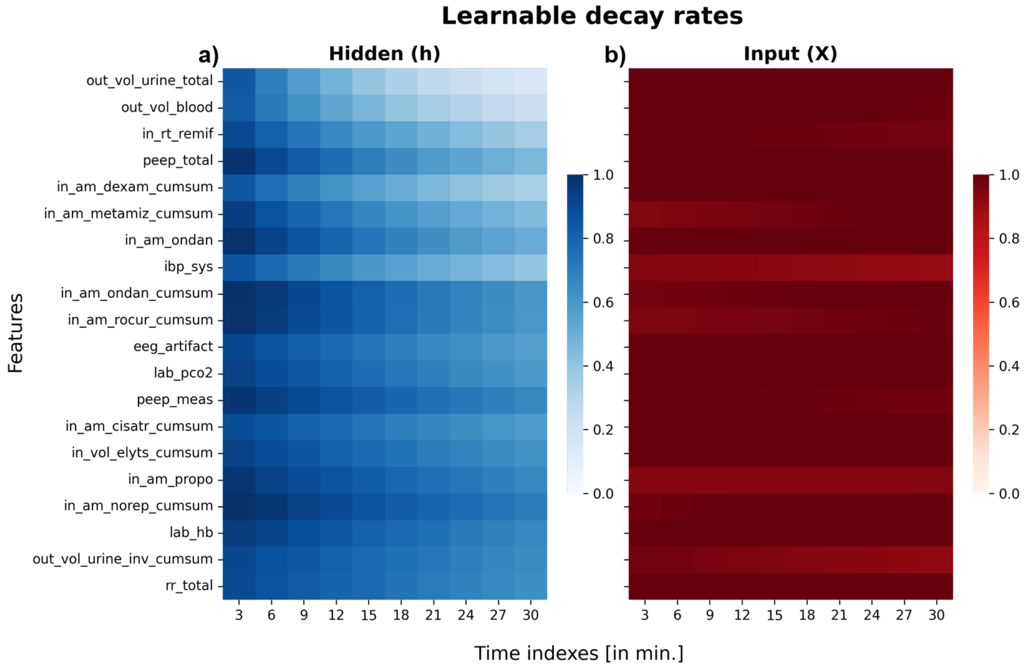

Decay rates learned by GRU-D model either with respect to hidden states (h) (blue-colorized) or to the model's input (X) (red-colorized graph) across time indexes (minutes) shown in **a** and **b**. Figure displays top 20 time-dynamic features with highest standard deviation of decay rates across time indexes.

We evaluated the trained GRU-D model with a mean AUROC of 0.747 (95% CI [0.731, 0.751]) and a mean AUPRC of 0.282 (95% CI [0.271, 0.293]) (see Supplementary Figure 11). GRU-D performed better than our best trained MLP model but failed to outperform TRAPOD. We also retrieved γ_h as well as γ_X per feature expressing the highest standard deviation (std) across time steps (see Supplementary Figure 12). In general, we could observe more temporal signal in γ_h (mean std = 0.135), than in γ_X (mean std = 0.014). Model features encoding urine - (out_vol_urine_total) and blood output (out_vol_blood) as well as the rate of remifentanil (in_rt_remif) expressed γ_h-stds of 0.229, 0.200, and 0.181, respectively. Temporal availability of features like invasive blood pressure (ibp_sys), given medications (in_am_propo), and vital signs (rr_total) was also identified by our univariate analysis (see main manuscript) as important for the POD prediction task.

Supplementary Discussion 1

We found high levels of blood pressure towards the end of the surgery and reduced doses of the vasoconstructive drug norepinephrine to be positively correlated with POD. Increasing the doses of norepiniphrine usually results in elevated levels of blood pressure[40] raising the chance of suffering from POD. Elevated blood pressure might indicate surgical stress evidently associated with POD[41]. Hence, advising higher doses of vasoconstructive medication seems counterintuitive. Results show that clinical variables are highly dependent on each other and that we cannot differentiate between treatment indication and treatment effect. Previous studies identified the variability of intraoperative parameters to be associated with POD[26,42–44]. Hirsch et al.[45] found that variance in systolic blood pressure had significant effects on POD. Wang et al.[46] reported that both high and low mean blood pressure levels (deviating from 80 mmHg) were significantly associated with POD occurrence. In our data, non-invasive blood pressure had a considerable standard deviation of 22.05 mmHg for POD cases and 20.97 mmHg for cases without POD (see Supplementary Table 1). Significant blood loss, indicative of the invasiveness of surgery, was found as another predictor in our data and could be aggravated by high blood pressure[47]. Low blood pressure, on the other hand, could inhibit the brain's oxygen supply[48]. Xue et al. suggest actively maintaining high blood pressure values for improving POD outcomes of patients undergoing hip-replacements[49]. Either way, a clarification of the complex interactions, if at all possible, would require controlled experiments outside the scope of this study. We see our results as complementary with previous studies but we stress the need for taking temporal intraoperative dynamics into account when predicting clinical outcomes. Moreover, the reliance of our models on EEG derived features suggests close intraoperative EEG monitoring.

Supplementary Figure 13. Prediction window lengths

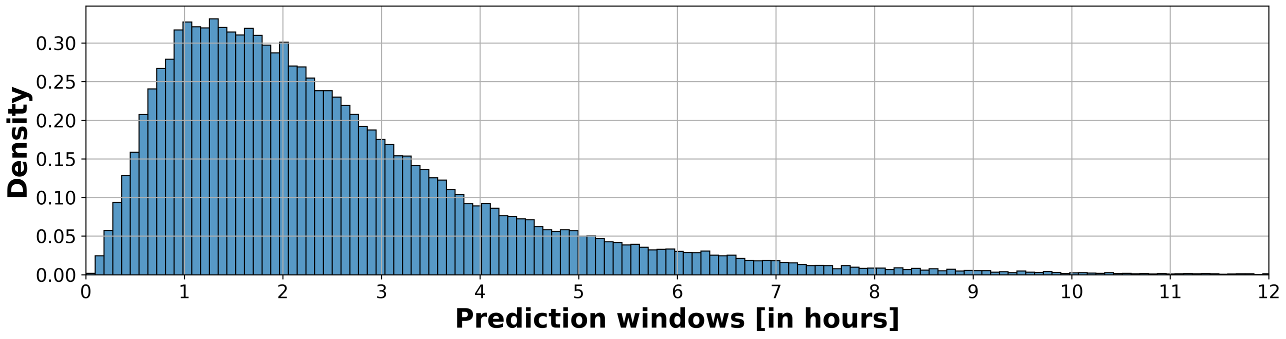

Distribution (#bins = 1000) of prediction windows (in hours) as durations between 30 min after start of intraoperative phase and the first Nu-DESC assessment. Prediction windows apply to trained transformers (TRANS_SEQ) with observation window [T_begin, 30] as the first itraoparative 30 min.

Supplementary Discussion 2

For considerations regarding a potential clinical model application, we retreived prediction windows for the included 72,100 surgeries as shown in Supplementary Figure 13. We draw conclusions based on our best performing TRAN_SEQ model ingesting [T_begin,30]. Prediction windows covered durations between [T_begin,30] and the first Nu-DESC assessment. Our model predicted over a median of 2.26 hours providing a sufficient amount of time for the application of POD preventing measurements.

Delirium guidelines recommend non-medical interventions as the reduction of stress, assistance with finding orientation, or the involvement of family members after surgery[41,50]. Vulnerable patients should be transferred to wards with extended monitoring after surgery like postoperative care units (PACUs) while the effect of medical interventions remained unclear[50]. The transfer from the operating room to the recovery room took in the median 6.24 minutes. In case of a positive ($Y = 1$) POD prediction by our model right at 30 minutes after surgery begin, the anesthesiologist could admit the high risk patient to an adjacent PACU instead of the recovery room. Noise reduction or the inclusion of relatives while waking up could also be a feasible cost-effective POD countermeasure for vulnerable patients.

References

1. Kuleshov, V. & Precup, D. Algorithms for multi-armed bandit problems. DOI: 10.48550/ARXIV.1402.6028 (2014). Publisher: arXiv Version Number: 1.

2. Li, L., Jamieson, K., DeSalvo, G., Rostamizadeh, A. & Talwalkar, A. Hyperband: A Novel Bandit-Based Approach to Hyperparameter Optimization. 52 (2016).
3. Wainer, J. & Cawley, G. Nested cross-validation when selecting classifiers is overzealous for most practical applications. *Expert. Syst. with Appl.* **182**, 115222, DOI: 10.1016/j.eswa.2021.115222 (2021).
4. Yu, Y., Si, X., Hu, C. & Zhang, J. A Review of Recurrent Neural Networks: LSTM Cells and Network Architectures. *Neural Comput.* **31**, 1235–1270, DOI: 10.1162/neco_a_01199 (2019).
5. Hochreiter, S. & Schmidhuber, J. Long Short-Term Memory. *Neural Comput.* **9**, 1735–1780, DOI: 10.1162/neco.1997.9.8.1735 (1997).
6. Hochreiter, S. The Vanishing Gradient Problem During Learning Recurrent Neural Nets and Problem Solutions. *Int. J. Uncertainty, Fuzziness Knowledge-Based Syst.* **06**, 107–116, DOI: 10.1142/S0218488598000094 (1998).
7. Sherstinsky, A. Fundamentals of Recurrent Neural Network (RNN) and Long Short-Term Memory (LSTM) network. *Phys. D: Nonlinear Phenom.* **404**, 132306, DOI: 10.1016/j.physd.2019.132306 (2020).
8. Dubey, S. R., Singh, S. K. & Chaudhuri, B. B. Activation functions in deep learning: A comprehensive survey and benchmark. *Neurocomputing* **503**, 92–108, DOI: 10.1016/j.neucom.2022.06.111 (2022).
9. Chung, J., Gulcehre, C., Cho, K. & Bengio, Y. Empirical evaluation of gated recurrent neural networks on sequence modeling. *arXiv preprint arXiv:1412.3555* (2014).
10. Vaswani, A. *et al.* Attention Is All You Need. DOI: 10.48550/ARXIV.1706.03762 (2017). Publisher: arXiv Version Number: 5.
11. Bahdanau, D., Cho, K. & Bengio, Y. Neural Machine Translation by Jointly Learning to Align and Translate. DOI: 10.48550/ARXIV.1409.0473 (2014). Publisher: arXiv Version Number: 7.
12. Aitken, K., Ramasesh, V. V., Cao, Y. & Maheswaranathan, N. Understanding How Encoder-Decoder Architectures Attend. DOI: 10.48550/ARXIV.2110.15253 (2021). Publisher: arXiv Version Number: 1.
13. Wen, Q. *et al.* Transformers in Time Series: A Survey (2023). ArXiv:2202.07125 [cs, eess, stat].
14. Chen, P.-C. *et al.* A Simple and Effective Positional Encoding for Transformers. DOI: 10.48550/ARXIV.2104.08698 (2021). Publisher: arXiv Version Number: 2.
15. Brown, T. B. *et al.* Language Models are Few-Shot Learners. DOI: 10.48550/ARXIV.2005.14165 (2020). Publisher: arXiv Version Number: 4.
16. Devlin, J., Chang, M.-W., Lee, K. & Toutanova, K. BERT: Pre-training of Deep Bidirectional Transformers for Language Understanding. DOI: 10.48550/ARXIV.1810.04805 (2018). Publisher: arXiv Version Number: 2.
17. Paszke, A. *et al.* PyTorch: An Imperative Style, High-Performance Deep Learning Library (2019). ArXiv:1912.01703 [cs, stat].
18. Gal, Y. & Ghahramani, Z. A Theoretically Grounded Application of Dropout in Recurrent Neural Networks. DOI: 10.48550/ARXIV.1512.05287 (2015). Publisher: arXiv Version Number: 5.
19. Ruppert, M. M. *et al.* ICU Delirium-Prediction Models: A Systematic Review. *Critical Care Explor.* **2**, e0296, DOI: 10/gmf4ck (2020).
20. Tan, M. C. *et al.* Incidence and Predictors of Post-Cardiotomy Delirium. *The Am. J. Geriatr. Psychiatry* **16**, 575–583, DOI: 10.1097/JGP.0b013e318172b418 (2008).
21. Witlox, J. *et al.* Delirium in Elderly Patients and the Risk of Postdischarge Mortality, Institutionalization, and Dementia: A Meta-analysis. *JAMA* **304**, 443, DOI: 10.1001/jama.2010.1013 (2010).
22. Iamaroon, A. *et al.* Incidence of and risk factors for postoperative delirium in older adult patients undergoing noncardiac surgery: a prospective study. *BMC Geriatr.* **20**, 40, DOI: 10.1186/s12877-020-1449-8 (2020).
23. Lopez-del Rio, A., Martin, M., Perera-Lluna, A. & Saidi, R. Effect of sequence padding on the performance of deep learning models in archaeal protein functional prediction. *Sci. Reports* **10**, 14634, DOI: 10.1038/s41598-020-71450-8 (2020).
24. Wassenaar, A. *et al.* Multinational development and validation of an early prediction model for delirium in ICU patients. *Intensive Care Medicine* **41**, 1048–1056, DOI: 10/f3pd3w (2015).
25. van den Boogaard, M. *et al.* Recalibration of the delirium prediction model for ICU patients (PRE-DELIRIC): a multinational observational study. *Intensive Care Medicine* **40**, 361–369, DOI: 10/f25hxv (2014).

26. Kim, M. Y., Park, U. J., Kim, H. T. & Cho, W. H. DELirium Prediction Based on Hospital Information (Delphi) in General Surgery Patients. *Medicine* **95**, e3072, DOI: 10/gndpjd (2016).

27. Vreeswijk, R., Kalisvaart, I., Maier, A. B. & Kalisvaart, K. J. Development and validation of the delirium risk assessment score (DRAS). *Eur. Geriatr. Medicine* **11**, 307–314, DOI: 10.1007/s41999-019-00287-w (2020).

28. Zucchelli, A. *et al.* Development and validation of a delirium risk assessment tool in older patients admitted to the Emergency Department Observation Unit. *Aging Clin. Exp. Res.* **33**, 2753–2758, DOI: 10.1007/s40520-021-01792-4 (2021).

29. Xing, H., Xiang, D., Li, Y., Ji, X. & Xie, G. Preoperative prognostic nutritional index predicts postoperative delirium in elderly patients after hip fracture surgery. *Psychogeriatrics* **20**, 487–494, DOI: 10.1111/psyg.12511 (2020).

30. PAWEL Study group *et al.* Patient safety, cost-effectiveness, and quality of life: reduction of delirium risk and postoperative cognitive dysfunction after elective procedures in older adults—study protocol for a stepped-wedge cluster randomized trial (PAWEL Study). *Trials* **20**, 71, DOI: 10/gjgcvd (2019).

31. Davoudi, A. *et al.* Delirium Prediction using Machine Learning Models on Preoperative Electronic Health Records Data. *Proceedings. IEEE Int. Symp. on Bioinforma. Bioeng.* **2017**, 568–573, DOI: 10/gmf4f9 (2017).

32. Lee, S., Mueller, B., Street, W. N. & Carnahan, R. M. Machine learning algorithm to predict delirium from emergency department data. preprint, Emergency Medicine (2021). DOI: 10.1101/2021.02.19.21251956.

33. Racine, A. M. *et al.* Machine Learning to Develop and Internally Validate a Predictive Model for Post-operative Delirium in a Prospective, Observational Clinical Cohort Study of Older Surgical Patients. *J. Gen. Intern. Medicine* DOI: 10/ghh53b (2020).

34. Giesa, N. https://github.com/ngiesa (2023).

35. Che, Z., Purushotham, S., Cho, K., Sontag, D. & Liu, Y. Recurrent neural networks for multivariate time series with missing values. *Sci. reports* **8**, 6085 (2018).

36. Groenwold, R. H. H. Informative missingness in electronic health record systems: the curse of knowing. *Diagn. Progn. Res.* **4**, 8, DOI: 10.1186/s41512-020-00077-0 (2020).

37. Lachin, J. M. Fallacies of last observation carried forward analyses. *Clin. Trials* **13**, 161–168, DOI: 10.1177/1740774515602688 (2016).

38. Lin, T.-Y., Goyal, P., Girshick, R., He, K. & Dollár, P. Focal Loss for Dense Object Detection. DOI: 10.48550/ARXIV.1708.02002 (2017). Publisher: arXiv Version Number: 2.

39. Yao, Y., Rosasco, L. & Caponnetto, A. On Early Stopping in Gradient Descent Learning. *Constr. Approx.* **26**, 289–315, DOI: 10.1007/s00365-006-0663-2 (2007).

40. Foulon, P. & De Backer, D. The hemodynamic effects of norepinephrine: far more than an increase in blood pressure! *Annals Transl. Medicine* **6**, S25–S25, DOI: 10.21037/atm.2018.09.27 (2018).

41. Aldecoa, C. *et al.* European Society of Anaesthesiology evidence-based and consensus-based guideline on postoperative delirium. *Eur. J. Anaesthesiol.* **34**, 192–214, DOI: 10/f925br (2017).

42. Zhang, C. *et al.* Association between intraoperative mean arterial pressure variability and postoperative delirium after hip fracture surgery: a retrospective cohort study. *BMC Geriatr.* **23**, 735, DOI: 10.1186/s12877-023-04425-9 (2023).

43. Fu, Z. *et al.* Admission Systolic Blood Pressure Predicts Post-Operative Delirium of Acute Aortic Dissection Patients in the Intensive Care Unit. *Int. J. Gen. Medicine* **Volume 14**, 5939–5948, DOI: 10.2147/IJGM.S329689 (2021).

44. Ooms, M. *et al.* Influence of perioperative blood pressure regulation on postoperative delirium in patients undergoing head and neck free flap reconstruction. *Eur. J. Med. Res.* **28**, 365, DOI: 10.1186/s40001-023-01367-1 (2023).

45. Hirsch, J., DePalma, G., Tsai, T., Sands, L. & Leung, J. Impact of intraoperative hypotension and blood pressure fluctuations on early postoperative delirium after non-cardiac surgery. *Br. journal anaesthesia* **115**, 418–426 (2015).

46. Wang, N.-Y., Hirao, A. & Sieber, F. Association between intraoperative blood pressure and postoperative delirium in elderly hip fracture patients. *PloS one* **10**, e0123892 (2015).

47. Pacagnella, R. C. *et al.* A Systematic Review of the Relationship between Blood Loss and Clinical Signs. *PLoS ONE* **8**, e57594, DOI: 10.1371/journal.pone.0057594 (2013).

48. Ringer, S. *et al.* Effects of moderate and severe arterial hypotension on intracerebral perfusion and brain tissue oxygenation in piglets. *Br. J. Anaesth.* **121**, 1308–1315, DOI: 10.1016/j.bja.2018.07.041 (2018).

49. Xu, X. *et al.* Effects of different bp management strategies on postoperative delirium in elderly patients undergoing hip replacement: a single center randomized controlled trial. *J. clinical anesthesia* **62**, 109730 (2020).

50. Aldecoa, C. *et al.* Update of the European Society of Anaesthesiology and Intensive Care Medicine evidence-based and consensus-based guideline on postoperative delirium in adult patients. *Eur. J. Anaesthesiol.* **41**, 81–108, DOI: 10.1097/EJA.0000000000001876 (2024).
