# Supplementary material for: Applying a transformer architecture to intraoperative temporal dynamics improves the prediction of postoperative delirium
Source: Commun Med (Lond). 2024 Nov 27;4:251. doi: 10.1038/s43856-024-00681-x (PMC11603037; doi:10.1038/s43856-024-00681-x)
Supplement: Supplementary file 3 — Description of Additional Supplementary File [file 43856_2024_681_MOESM3_ESM.pdf]

Description Of Additional Supplementary File

File name: Supplementary Data 1
Description: Features that were extracted from clinical information systems. We selected 148 features out of 197 extracted ones due to the availability during the intraoperative phase.

File name: Supplementary Data 2
Description: The missingness is shown per feature. We calculated the number of patients having no recorded longitudinal data at all for one feature from all patients (FETS). In addition, we calculated the sparsitiy per time series and feature as the fraction of missing time points from all time points. The results were mean averaged across patients (see Methods).

File name: Supplementary Data 3
Description: ICD and OPS codes encodings.

File name: Supplementary Data 4
Description: Descriptive statistics of features for initial train and test split per sampling interval.

File name: Supplementary Data 5
Description: Feature encodings for baseline models. Numeric feature values were median-aggregated intraoperatively.

File name: Supplementary Data 6
Description: Hyperparameter sets evaluated via 3x3 fold nested cross-validation.

File name: Supplementary Data 7
Description: Checklist for TRIPOD guidelines.

File name: Supplementary Data 8
Description: Spearman's correlation coefficient with FDR corrected p-values applied for mean values aggregated for time windows residing inside the intraoperative time phase [T_begin, T_end].

File name: Supplementary Data 9
Description: Evaluation metrics on 1000x bootstrapped testing set for model variants and observation windows. Precision is calculated for 0.8 recall. Sensitivities and specificities are retreived for the threshold where their sums maximize. Random classification levels are 0.5 and 0.9 for AUROC and AUPRC respectively. Results are reported as mean (95%-CI).
