# Supplementary material for: Applying a transformer architecture to intraoperative temporal dynamics improves the prediction of postoperative delirium
Source: Commun Med (Lond). 2024 Nov 27;4:251. doi: 10.1038/s43856-024-00681-x (PMC11603037; doi:10.1038/s43856-024-00681-x)
Supplement: Supplementary file 13 — Reporting Summary [file 43856_2024_681_MOESM13_ESM.pdf]

nature portfolio

Corresponding author(s): Niklas Giesa

Last updated by author(s): Oct 10, 2024

Reporting Summary

Nature Portfolio wishes to improve the reproducibility of the work that we publish. This form provides structure for consistency and transparency in reporting. For further information on Nature Portfolio policies, see our Editorial Policies and the Editorial Policy Checklist.

Statistics

For all statistical analyses, confirm that the following items are present in the figure legend, table legend, main text, or Methods section.

n/a	Confirmed	
☐	☒	The exact sample size (*n*) for each experimental group/condition, given as a discrete number and unit of measurement
☐	☒	A statement on whether measurements were taken from distinct samples or whether the same sample was measured repeatedly
☐	☒	The statistical test(s) used AND whether they are one- or two-sided<br>*Only common tests should be described solely by name; describe more complex techniques in the Methods section.*
☐	☒	A description of all covariates tested
☐	☒	A description of any assumptions or corrections, such as tests of normality and adjustment for multiple comparisons
☐	☒	A full description of the statistical parameters including central tendency (e.g. means) or other basic estimates (e.g. regression coefficient) AND variation (e.g. standard deviation) or associated estimates of uncertainty (e.g. confidence intervals)
☐	☒	For null hypothesis testing, the test statistic (e.g. *F*, *t*, *r*) with confidence intervals, effect sizes, degrees of freedom and *P* value noted<br>*Give P values as exact values whenever suitable.*
☒	☐	For Bayesian analysis, information on the choice of priors and Markov chain Monte Carlo settings
☒	☐	For hierarchical and complex designs, identification of the appropriate level for tests and full reporting of outcomes
☐	☒	Estimates of effect sizes (e.g. Cohen's *d*, Pearson's *r*), indicating how they were calculated

Our web collection on statistics for biologists contains articles on many of the points above.

Software and code

Policy information about availability of computer code

Data collection	Data were initially extracted via the data engineering team using NiFi and HDFS integrated into the Hadoop infrastructure. Source databases comprised two MSSQL instances. Afterwards, data was stored in a central data lake and accessed by NG. The accessed data was validated with the clinical front-end system Copra 6 by executing SQL queries against the HDFS via Impala and Hue. After validation, POD specific data were stored locally and transferred with Python 3.6 using sql alchemy and odbc. Python scripts were used for preprocessing residing on the local data instance. See requirements.txt on https://github.com/ngiesa/TRAPOD.
Data analysis	Data was analyzed with the Python packages: numpy, statsmodels, pandas, sklearn, and pytorch. For concrete version control information see the requirements.txt on https://github.com/ngiesa/TRAPOD.

For manuscripts utilizing custom algorithms or software that are central to the research but not yet described in published literature, software must be made available to editors and reviewers. We strongly encourage code deposition in a community repository (e.g. GitHub). See the Nature Portfolio guidelines for submitting code & software for further information.

Data

Policy information about availability of data

All manuscripts must include a data availability statement. This statement should provide the following information, where applicable:

- Accession codes, unique identifiers, or web links for publicly available datasets
- A description of any restrictions on data availability
- For clinical datasets or third party data, please ensure that the statement adheres to our policy

Code and trained models can be found in our public GitHub https://github.com/ngiesa/TRAPOD including usage instructions. Data sharing is not possible due to data privacy and protection regulations at our clinical institution. We uploaded all descriptive information on the public data repository Dryad 10.5061/dryad.bvq83bkhv (Reviewer sharing link: https://datadryad.org/stash/share/JDGY_rqv4nmQodtW0FX5UOzEv93726JABMa0nVB2ZbM)

Human research participants

Policy information about studies involving human research participants and Sex and Gender in Research.

Reporting on sex and gender	We collected gender information since patients actively validated related information during the anesthesia consent meeting in their electronic file. We could not integrate additional information during the anesthesia consent meeting since we had no active role in the clinical routine. We used this single information and did not differentiate further. Our clinical institution is currently implementing processes to obtain more specific data points regarding gender and sex.
Population characteristics	The study cohort consisted of 60,436 patients undergoing a total of 72,100 surgeries within 68,983 distinct hospital stays with a POD prevalence of 9.38%, 8.27%, and 8.47%, respectively (see Figure A.1 in Supplement A). The presence of POD was defined as at least one Nu-DESC ≥ 1 measured in the recovery room (see Methods). On average, patients experiencing POD had prolonged stays in the hospital (11.45 ± 7.63 vs. 8.31 ± 5.78 days) and in the recovery room (2.51 ± 7.63 vs. 2.03 ± 2.63hours). They also underwent longer anaesthesia (4.65 ± 1.36 vs. 2.99 ± 1.24 hours). While the mean age was higher for POD positive patients (60.28 ± 18.88 vs. 52.93 ± 18.22 years), there was no pronounced difference in the gender distribution (51.33 vs. 53.60% female). Patients experiencing POD seemed to be more frail with a weaker physical preoperative status (see Table A.1 in Supplement A). Most common comorbidities were dementia, Parkinson, stroke or CHD. Surgeries were also more performed in abdominal, otorhinolary, and endocrine domains than in others.
Recruitment	There was no specific patient recruiting process due to the inclusion of electronic health records. We included all adult (older than 18 years) patients that were evaluated with at least one Nu-DESC score in the recovery room from 2017 – 2020 and underwent non-cardiac and non-craniotomy procedures. We also just included surgeries that were at least 30 min and max. 12h long. Potential biases are: documentation bias in IT-systems, exclusion of vulnerable patients that underwent invasive surgical procedures, omitting of pediatric patients. We validated clinical records with our front-end system to avoid documentation bias. We also believe that our analysis was more relevant on patients with less invasive measurements due to low incidences. Investigations with pediatric patients should be performed separately.
Ethics oversight	This work was approved by the local institutional review board of the Charité - Universitätsmedizin Berlin under the internal ethics approval number EA4/254/21.

Note that full information on the approval of the study protocol must also be provided in the manuscript.

Field-specific reporting

Please select the one below that is the best fit for your research. If you are not sure, read the appropriate sections before making your selection.

☒ Life sciences ☐ Behavioural & social sciences ☐ Ecological, evolutionary & environmental sciences

For a reference copy of the document with all sections, see nature.com/documents/nr-reporting-summary-flat.pdf

Life sciences study design

All studies must disclose on these points even when the disclosure is negative.

Sample size	The study cohort consisted of 60,436 patients undergoing a total of 72,100 surgeries within 68,983 distinct hospital stays with a POD prevalence of 9.38%, 8.27%, and 8.47%, respectively (see Figure A.1 in Supplement A).
Data exclusions	Data exclusions were made as follows: Adult patients admitted 2017-2020 (116,534 patients, 150,174 hospital stays, 172,624 surgeries) -> Non-cardiac and non-craniotomy surgeries (170,040 surgeries), Anesthesia between 0.5 and 12h (168,032 surgeries), Surgeries with recover room admission (96,715 surgeries) -> 72,100 surgeries with Nu-DESC evaluation
Replication	We wrote reusable code and published our best performing model variants to replicate our findings. We also estimated uncertainty on a

Replication	bootstrapped and resampled testing set using confidence intervals for estmations.
Randomization	We randomly divided our data into train and testing sets. We controlled for covariates by using mixed-linear effect models integrating covariates inside the model. Our univariate analysis was independent from these effects. Prediction models have learned with all covariates that were included as model predictors.
Blinding	Blinding does not apply directly due to the extraction of electronic health records. We have not seen any patients physically that were included into our study. The bias of any relation between providing health care and performing this study can be seen as non-existent.

Reporting for specific materials, systems and methods

We require information from authors about some types of materials, experimental systems and methods used in many studies. Here, indicate whether each material, system or method listed is relevant to your study. If you are not sure if a list item applies to your research, read the appropriate section before selecting a response.

Materials & experimental systems

n/a	Involved in the study
☒	☐ Antibodies
☒	☐ Eukaryotic cell lines
☒	☐ Palaeontology and archaeology
☒	☐ Animals and other organisms
☐	☒ Clinical data
☒	☐ Dual use research of concern

Methods

n/a	Involved in the study
☒	☐ ChIP-seq
☒	☐ Flow cytometry
☒	☐ MRI-based neuroimaging

Clinical data

Policy information about clinical studies

All manuscripts should comply with the ICMJE guidelines for publication of clinical research and a completed CONSORT checklist must be included with all submissions.

Clinical trial registration	We did not performed a clinical trial. This work was approved by the local institutional review board of the Charité - Universitätsmedizin Berlin under the internal ethics approval number EA4/254/21
Study protocol	The study protocol does not apply for our work relying on retropective health records.
Data collection	Data was collected in our clinical information systems between 2017 and 2020 at three hospital sides.
Outcomes	Postoperative delirium (POD) (and the target variable Y) was defined with the nursing delirium screening scale (Nu-DESC). Nu-DESC assessment in the recovery room for POD diagnosis has been established as a standard procedure at our clinical institution over the past years. The Nu-DESC test assigns 0-2 points to each of the following five categories: disorientation, inappropriate behaviour, inappropriate communication, hallucinations, and psycho-motor retardation. The Nu-DESC score is the sum of all points across categories. If a patient was assessed with at least one Nu-DESC point, the corresponding surgery was labeled POD positive. If all Nu-DESC categories were scored zero, the surgery was labeled POD negative. We applied a threshold of one as suggested by clinical guidelines, due to the resulting enhancement in sensitivity, aiming at detecting highly vulnerable patients. If all Nu-DESC scores = 0, then Y=0, else Y=1.
